# Regulation Mediated by *N*-Acyl Homoserine Lactone Quorum Sensing Signals in the *Rhizobium*-Legume Symbiosis

**DOI:** 10.3390/genes9050263

**Published:** 2018-05-18

**Authors:** Nieves Calatrava-Morales, Matthew McIntosh, María J. Soto

**Affiliations:** 1 Departamento de Microbiología del Suelo y Sistemas Simbióticos, Estación Experimental del Zaidín, CSIC; Granada 18008, Spain; nieves.calatrava@eez.csic.es; 2 Institut für Mikrobiologie und Molekularbiologie, Universität Giessen, 35392 Giessen, Germany; matthew.mcintosh@mikro.bio.uni-giessen.de

**Keywords:** *Sinorhizobium meliloti*, long-chain AHL, exopolysaccharides, motility, symbiosis, regulation, interkingdom communication, LuxR solos

## Abstract

Soil-dwelling bacteria collectively referred to as rhizobia synthesize and perceive *N*-acyl-homoserine lactone (AHL) signals to regulate gene expression in a population density-dependent manner. AHL-mediated signaling in these bacteria regulates several functions which are important for the establishment of nitrogen-fixing symbiosis with legume plants. Moreover, rhizobial AHL act as interkingdom signals triggering plant responses that impact the plant-bacteria interaction. Both the regulatory mechanisms that control AHL synthesis in rhizobia and the set of bacterial genes and associated traits under quorum sensing (QS) control vary greatly among the rhizobial species. In this article, we focus on the well-known QS system of the alfalfa symbiont *Sinorhizobium (Ensifer) meliloti*. Bacterial genes, environmental factors and transcriptional and posttranscriptional regulatory mechanisms that control AHL production in this *Rhizobium*, as well as the effects of the signaling molecule on bacterial phenotypes and plant responses will be reviewed. Current knowledge of *S. meliloti* QS will be compared with that of other rhizobia. Finally, participation of the legume host in QS by interfering with rhizobial AHL perception through the production of molecular mimics will also be addressed.

## 1. Introduction

*Sinorhizobium meliloti* is a soil-dwelling α-proteobacterium that can exist in a free-living state or can establish nitrogen-fixing symbiosis with legume plants belonging to the genera *Medicago*, *Melilotus* and *Trigonella*. In this symbiosis, bacteria invade the legume root leading to the formation of new organs called nodules, in which specialized forms of the microsymbiont fix atmospheric nitrogen that is transferred to the plant in exchange for carbohydrates and a protected niche. The formation of symbiotic nitrogen-fixing nodules requires a continuous molecular dialogue that co-ordinates two developmental processes: bacterial infection initiated in the epidermis and nodule organogenesis that takes place in the root cortex [1,2,3]. To initiate this interaction, legume plants secrete a variety of compounds that attract the bacteria toward their roots and activate the production of a lipochitooligosaccharide signal molecule known as the Nod factor [4,5]. Nod factor perception in the plant root induces a cascade of signaling events, leading to root hair curling and cortical cell division necessary for root nodule formation. Root hair deformation entraps bacteria that initiate invasion of roots through a tube-like structure called the infection thread [6]. This process requires production of symbiotically important exopolysaccharides (EPS): succinoglycan (EPS I) or galactoglucan (EPS II) [4,7,8,9,10]. The infection threads grow towards the emergent nodules and ramify within the nodule tissue. Finally, the bacteria are released into plant cells in an endocytotic process and maintained in membrane-bound compartments called symbiosomes. Bacteria stop dividing and differentiate into morphologically new forms called bacteroids, which are able to perform the reduction of atmospheric dinitrogen into nitrogenated compounds for the plant [11,12,13,14].

The rhizosphere, i.e. the zone of soil that surrounds and is influenced by plant roots, typically provides suitable growth conditions for bacteria. Following bacterial colonization of plant roots, an increase in the bacterial population density triggers the onset of quorum sensing (QS). QS is a cell-to-cell signaling mechanism that allows rhizobia to collectively modify behaviors, some of which are crucial for the interaction with its host. Functions regulated by QS in legume-nodulating rhizobia include plasmid transfer, surface polysaccharides, motility, adaptation to stationary phase of growth and symbiotic interactions (reviewed in [4,15,16,17]). Different chemical signals have been identified to participate in QS in bacteria [18,19]. In rhizobia, QS usually relies on the synthesis and detection of *N*-acyl-homoserine lactones (AHL) [16,17], although some exceptions are known, such as the unusual branched-chain isovaleryl-homoserine lactone and bradyoxetin from the soybean symbiont *Bradyrhizobium japonicum* [20,21], the cinnamoyl-homoserine lactone from stem-nodulating photosynthetic bradyrhizobia [22], or the response of *S. meliloti* to the so-called autoinducer-2 (AI-2) [23]. Given the impact that AHLs have in several phenomena related to the development of an efficient *Rhizobium*-legume symbiosis, rhizobial AHLs can be considered symbiotically-important signals, although in some cases the contribution of AHLs are subtle and may reflect AHL-related differences in metabolism rather than direct regulatory mechanisms. Compared to other genera, rhizobia produce the greatest variety of AHLs. Here, we will review recent findings concerning QS regulation in the model bacterium *S. meliloti* and the role played by AHLs in intercellular and interkingdom communication. The knowledge of QS-related aspects in other rhizobia will be compared.

## 2. Quorum Sensing Genes in *Sinorhizobium meliloti*

**Figure 1:**
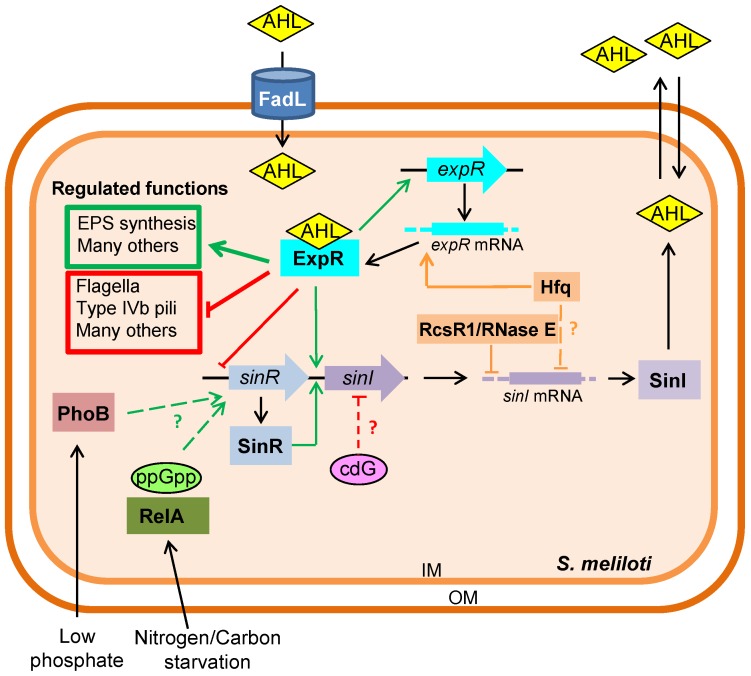
Model of transcriptional and post-transcriptional regulation of quorum sensing (QS) in *Sinorhizobium meliloti*. Transcriptional autoregulation of the ExpR/Sin system is shown. *N*-acyl-homoserine lactone (AHL)-activated ExpR induces *sinI* and inhibits *sinR*. AHL-activated ExpR activates *expR* expression weakly. Transcriptional activation of *sinR* expression mediated by PhoB and RelA in response to nutrient limitation is also represented. Cyclic diguanylate (cdG) negatively affects *sinI* expression and AHL synthesis by an as yet unknown mechanism which is *expR* independent. Post-transcriptional regulation of the *sinI* transcript mediated by the RNA chaperone Hfq, RNAse E and the small RNA RcsR1 is shown in orange. See text for more details. The protein FadL that facilitates sensing of the long-chain AHLs produced by *S. meliloti* is represented. Genes are represented by wide arrows, proteins by rectangles, and second messenger by ovals. Green arrows represent activation; red flat-ended lines indicate repression; orange arrows and flat-ended lines indicate positive and negative post-transcriptional regulations, respectively. Question mark indicates that the mechanism of regulation is unknown. AHL: *N*-acyl homoserine lactone; IM: inner membrane; OM: outer membrane.

Most of the knowledge of QS in *S. meliloti* has been obtained using the reference strain Rm1021. In this bacterium, three genes, *sinI*, *sinR*, and *expR*, are essential for QS regulation (Figure 1). *sinI* encodes a LuxI-type synthase that catalyzes AHL production from two substrates: *S*-adenosyl-L-methionine (SAM), which provides the homoserine lactone ring moiety, and an appropriate acyl-acyl carrier protein (acyl-ACP) from fatty acid biosynthesis, which is the precursor of the fatty acyl side chain. SinI is responsible for the production of diverse long-chain AHLs, including C12-HL, C14-HL, 3-oxo-C14-HL, C16-HL, 3-oxo-C16-HL, C16:1-HL, 3-oxo-C16:1-HL and C18-HL, with 3-oxo-C16:1-HL being the most abundant in laboratory cultures [24,25,26]. Adjacent and upstream of the chromosomal *sinI* gene is *sinR*, which encodes a LuxR-type transcription regulator that controls *sinI* expression but whose activity is not affected by AHLs [24,27,28]. Instead, the product of the *expR* gene is the major regulator of AHL-controlled gene expression in *S. meliloti* [26,29,30]. ExpR is an orphan LuxR-type transcriptional regulator or “LuxR solo”, i.e. a LuxR-type receptor that is not paired with a cognate AHL synthase [31,32]. Expression of *sinI* is strongly enhanced by ExpR in the presence of AHLs, resulting in a positive feedback. This positive feedback mechanism is a hallmark feature of the classical QS system [33]. As will be explained in detail in Section 3.1.1 of this review, ExpR also represses transcription of *sinR* at high AHL concentrations, which leads to negative feedback regulation of *sinI* [34] (Figure 1).

Compared to other orphan LuxR-type proteins, ExpR is a highly versatile regulator with different regulatory capabilities. More than 500 genes have been identified as differentially expressed by ExpR [30] and over 30 DNA binding sites have been biochemically identified [28,35]. Although ExpR can act as an AHL-independent regulator of genes involved in metabolic processes, the presence of AHLs is necessary for most of the regulatory targets of ExpR, including the two best-known functions controlled by QS in *S. meliloti*: activation of EPS synthesis and repression of flagella production (see Section 5; Figure 1). The location of the ExpR-binding site within the promoter region, e.g., upstream or downstream of the −35/−10 promoter elements, determines whether ExpR/AHL activates or represses transcription. In addition, transcriptional activity of QS-regulated genes is dependent upon the concentration of AHLs [28]. This property allows for a temporal expression program in which different target promoters have different AHL concentration thresholds for their activation or repression. As an example, genes required for EPS production in *S. meliloti* are activated at lower concentrations of AHLs than those required for the repression of flagella-associated genes [28,30].

The *expR* gene is disrupted by an insertion element in the widely studied *S. meliloti* laboratory strains Rm1021 and Rm2011 and its functional restoration causes a moderate (3-fold) increase in production of the symbiotically important EPS succinoglycan (EPS I) and an especially strong increase in galactoglucan (EPS II) production, which confers an extremely mucoid phenotype to the colonies [36,37,38,39]. Remarkably, the dry phenotype exhibited by various laboratory strains of *S. meliloti* from multiple independent isolates has been accredited to spontaneous mutations at the *expR* locus [36,40]. It was found that *expR* and therefore QS is rapidly inactivated during routine laboratory cultivation of *S. meliloti* strains. This inactivation confers a selective advantage due to ExpR-dependent restraint of bacterial growth whose mechanism is unknown [40]. Interestingly, ExpR and QS are stable in natural environments, suggesting that maintaining an intact QS system confers bacterial benefits that remain to be discovered. Despite the loss of ExpR, these mutant strains still produce significant levels of AHLs [24,25], but the presence of these AHLs affects the expression of only few genes [29,38] via an unknown mechanism.

In addition to ExpR and SinR, the *S. meliloti* Rm1021 genome potentially codes for at least eight additional LuxR-type proteins. These are SMc00658, SMc00877, SMc00878, SMc03015 (VisN), SMc03016 (VisR), SMc04032 (NesR) and SMc03150. In addition, many other open reading frames (ORFs) could code for proteins harboring LuxR-domains, but differ from the standard LuxR-like proteins, e.g., SMc01630 which has a larger size. Thus, the number of genes coding for LuxR-type proteins could be higher. For example, several species of the Gammaproteobacterium *Photorhabdus* are reported to contain up to 40 *luxR*-like orphans [32]. All of the *luxR*-like genes in *S. meliloti*, with the exception of *sinR*, are not associated with a known AHL synthase-encoding locus, and therefore, as with ExpR, can be considered orphan LuxR homologs [31,41]. LuxR solos can respond to endogenous or exogenous AHLs, to endogenous signals which are not AHLs, or to low molecular weight plant compounds [41]. In *S. meliloti*, only ExpR is known to respond to AHLs as the effector molecule. For the other orphan LuxR-type proteins and SinR, the mechanisms and potential ligands involved in activating these transcriptional regulators remain to be discovered. Likewise, little is known about the biological role of most of these LuxR homologs. VisN and VisR are the master regulators of the *S. meliloti* flagellar regulon and are essential for the expression of flagellar and motility genes [42]. These are encoded within an operon whose promoter binds to and is repressed by the ExpR/AHL complex [43]. VisN and VisR are proposed to form a heterodimer and variability in their ligand-binding domains suggest that these proteins bind different effector molecules whose nature remains unknown [42]. SMc00878 and SMc00877 are highly homologous to AviR of *Agrobacterium vitis* [44] and the former seems to affect the denitrification pathway of *S. meliloti* [31]. In the case of *nesR*, its promoter also binds to the ExpR/AHL complex [28] and its expression and phenotypic analyses suggest that this regulator contributes to the bacterial adaptation to environmental stresses such as high osmotic conditions or nutrient deprivation, as well as to the bacterial ability to compete for nodule formation [45]. NesR belongs to a LuxR solo sub-family which is only found in plant associated bacteria, whose members do not bind AHLs but plant-produced compounds [46]. NesR is highly homologous to XccR and OryR of *Xanthomonas campestris* pv. campestris and *Xanthomonas oryzae* pv. oryzae, respectively. These orphan LuxR regulators respond to plant exudates and control bacterial virulence [47,48]. Whether a plant-produced or any other exogenous signal activates NesR warrants investigation.

In the *S. meliloti* strain Rm41, which also carries the ExpR/Sin QS system, the *traI* and *traR* genes were identified in a large plasmid (pRme41a) which is not present in Rm1021 [27] (Table 1). This second QS system in Rm41, which is involved in the production of AHLs unique to Rm41 (3-oxo-C8-HL), is located next to plasmid transfer genes. Such a scenario suggests that this system regulates transfer of the pRme41a upon reaching a quorum, as is the case for a homologous system in *Agrobacterium tumefaciens*.

The classical QS model assumes free diffusion of AHLs across the bacterial cell envelope. However, this was only demonstrated for the short-chain C4-HL. As mentioned earlier, *S. meliloti* produces long-chain AHLs. These molecules, in contrast to short-chain AHLs, cannot diffuse freely across the cell membranes. Recently, membrane vesicles have been shown to participate in the secretion of C16-HL produced by *Paracoccus* spp. and also guide its transport to target cells [49]. In *S. meliloti*, sensing of AHLs with acyl chains composed of 14 or more carbons is facilitated by the outer membrane protein FadL (Figure 1), a homolog of the *Escherichia coli* FadL long-chain fatty acid transporter [50]. In contrast to the *E. coli* FadL, *S. meliloti* FadL is not required for the uptake of oleic acid (C18:1) but participates in the uptake of palmitoleic acid (C16:1) and long-chain AHLs, especially C16:1-HL, increasing sensitivity to AHL levels external to the cell and accelerating the QS response. This AHL import ability by FadL homologs is conserved in rhizobial species belonging to the genera *Sinorhizobium*, *Rhizobium* and *Mesorhizobium*.

## 3. Regulatory Mechanisms Controlling AHL Synthesis in *Sinorhizobium meliloti*

The synthesis of AHL signals and expression of the corresponding QS-regulated genes in *S. meliloti* is controlled at multiple levels, which involve transcriptional as well as post-transcriptional mechanisms (Figure 1).

### 3.1. Transcriptional Mechanisms

#### 3.1.1. Autoregulation

The Sin system is subject to autoregulation through positive and negative feedback mechanisms which are mediated exclusively by ExpR, without the requirement of additional regulators [34]. The autoregulatory activity of ExpR depends upon the presence of AHLs which assist the strength of DNA binding [51]. AHL-activated ExpR induces *sinI* through a binding site upstream of *sinI* and inhibits *sinR* through another binding site upstream of *sinR*. Moreover, AHL-activated ExpR activates *expR* expression weakly. Considering that *sinI* expression is dependent on levels of SinR and that activation of *sinI* expression requires lower levels of AHL than *sinR* repression (1 nM *vs* 40 nM), AHL and ExpR levels will determine the dominance of the positive or negative feedback. Thus, at low AHL levels a positive feedback loop activates expression of *sinI*, leading to increased AHL levels. At high AHL levels, repression of *sinR* and therefore reduction of *sinI* expression takes place. As the AHL levels increase, the positive autoregulation by AHL-activated ExpR increases ExpR levels, which leads to a stronger repression of *sinR*, and the corresponding decrease in *sinI* expression and AHL levels.

#### 3.1.2. Nutrient Availability

Environmental factors including stress are known to regulate QS in rhizobia. As reported in different bacteria, nutrient availability also influences QS regulation in *S. meliloti*. Specifically, expression of *sinI* and *sinR* are positively regulated under low phosphate concentrations [34]. This regulation is mediated by PhoB, the response regulator of the two-component regulatory system PhoR/PhoB, which is responsible for the phosphate stress response in the alfalfa symbiont [52]. Therefore, this system links nutritional stress response to social behavior, probably increasing adaptability of bacteria. It is not known whether *sinR* is directly regulated by PhoB because a Pho box identified in the *sinR* promoter region is not required in the effect of PhoB on *sinR* expression.

Under sudden nutritional downshifts, the stringent response alarmone guanosine tetraphosphate (ppGpp) accumulates in *S. meliloti* cells, which leads to transcriptome remodeling. The protein RelA is responsible for the synthesis of ppGpp. Additional evidence for nutrient availability influencing AHL production was obtained after expression profiling performed on *S. meliloti* cells under nitrogen or carbon starvation. These studies revealed that *sinR* was induced in a RelA-dependent manner [53]. Likewise, in the bean symbiont *Rhizobium etli*, AHL accumulation is dependent on the *relA* homologue [54].

#### 3.1.3. Modulation of Quorum Sensing by Cyclic Diguanylate

QS and cyclic diguanylate (cdG)-dependent regulation are interconnected in several bacterial species. The most common mechanism is regulation of cdG biosynthesis or degradation by QS lowering cdG levels in the QS state [55]. In *S. meliloti*, QS does not seem to influence the total cdG content. In contrast, both native and elevated levels of cdG negatively affect expression of the AHL synthase gene *sinI* and accumulation of AHLs in the growth medium [56]. The factor mediating cdG-dependent negative regulation of the synthase gene is unknown. Since the cdG content was higher in exponential- than in stationary-phase *S. meliloti* cells [56], negative regulation of AHL biosynthesis by cdG may serve as a fine-tuning mechanism attenuating AHL accumulation in rapidly growing cells.

### 3.2. Post-Transcritpional Regulation

Regulation of QS in *S. meliloti* has been studied mainly at the level of transcription and little is known about factors acting post-transcriptionally. In other bacteria, small regulatory RNAs (sRNAs) have been found to regulate QS by controlling translation and mRNA levels of the LuxR-like transcriptional regulators or other QS-dependent genes [57,58]. Typically, sRNAs interact with mRNAs with the help of the RNA chaperone Hfq and influence the translation rate and/or half-life of the mRNA targets. Usually, this interaction acts as a repressor mechanism by inducing degradation of the sRNA-mRNA complex in an endoribonuclease E (RNase E)-dependent manner. RNase E plays a major role in mRNA decay in bacteria [59].

Interestingly, RNase E of *S. meliloti* affects the production of AHLs by interacting with a specific target sequence located in the 5’-untranslated region (UTR) of the autoinducer synthase encoding *sinI* mRNA (Figure 1). Independently of ExpR and Hfq, and without affecting *sinR* mRNA stability, overexpression of the RNase E coding sequence, *rne*, results in enhanced degradation of *sinI* transcript. Therefore, RNase E-dependent degradation of *sinI* mRNA from the 5’-end could be one of the steps mediating a high turn-over of *sinI* mRNA, which allows the Sin QS system to respond rapidly to changes in transcriptional control of AHL production [60].

Although not linked with RNase E activity, the RNA-binding protein Hfq contributes to the regulation of AHL production in *S. meliloti* (Figure 1). This regulation is exerted via both ExpR-dependent and -independent mechanisms [61]. In the *expR+* strain, the lack of Hfq leads to hyper-accumulation of QS signals at low population densities, and a sharp decrease in AHL accumulation in stationary phase. These results indicated that Hfq, directly or indirectly, exerts negative control over production or stability of AHLs at low population densities, and a positive control over AHL synthesis and/or accumulation, at high population densities. No regulatory effect of Hfq on *sinR* mRNA was observed. In contrast, it was demonstrated that Hfq influences *expR* and *sinI* expression. Whereas Hfq directly controls *expR* mRNA at higher population densities, the effects of Hfq on *sinI* at lower population densities seem to be mediated by other mechanisms [61].

More recently, a structurally conserved, trans-acting sRNA has been shown to directly interact with and to destabilize the *sinI* mRNA in *S. meliloti*. This sRNA, called RcsR1, is not regulated in a cell density-dependent manner, but shows similar expression profiles under salt and cold stress in several Rhizobiaceae members, which suggests a conserved role in response to environmental stress. Thus, RcsR1 links stress responses to QS in *S. meliloti* [62,63]. In *S. meliloti* the *sinI* mRNA steady-state levels and the AHL amounts diminish upon overexpression of *rcsR1*, a process that requires an intact *rne* gene, indicating that RcsR1 influences QS in an RNase E-dependent manner. Consistent with the RNase E effect on *sinI* mRNA degradation, RcsR1 operates by an Hfq-independent mechanism. Interestingly, although RcsR1 binds closely to the Shine-Dalgarno sequence and therefore prevents accessibility of RNase E to the 5’-UTR region of the *sinI* transcript, the sRNA increases the negative effect of RNase E on the *sinI* mRNA half-life. This can be explained by a block of ribosomal machinery by RcsR1 during translation leading to a better accessibility of RNase E cleavage sites in the *sinI* coding region [63].

Apart from the direct interaction between RcsR1 and *sinI* mRNA, three other targets have been verified: *phoR* encoding a sensor kinase for the response to phosphate limitation, *motE* encoding a chaperone for a periplasmic motility protein and *sm2011_c01420* encoding the anti-σE1 factor. As mentioned above, PhoB (the response regulator in the two-component system PhoR/PhoB) induces *sinR*. This implies that RcsR1 does not only influence QS in *S. meliloti* by direct interaction with *sinI*, but also indirectly through the PhoR/PhoB signaling cascade, balancing *sinI* expression under phosphate limiting conditions [63].

## 4. Quorum Sensing Regulation in Other Rhizobia

*S. meliloti* is a model organism among the rhizobia for studying QS regulation because of the presence of a single AHL-based system (ExpR/Sin). Nevertheless, investigations on QS regulatory systems in different rhizobia have shown that they are highly diverse and can control a broad range of bacterial functions (Table 1). In fact, even within a single rhizobial species, a different range of QS systems can be found among different isolates. This diversity suggests that there may be no unifying paradigm of what is controlled by QS in these bacteria [17]. One common characteristic is that all the AHL synthases and response regulators identified in rhizobia belong to the LuxI and LuxR protein families, respectively. Besides *S. meliloti*, the QS regulatory mechanisms of *Rhizobium leguminosarum* and *R. etli* have been well studied. Here, the most important characteristics of QS regulation in these two species will be summarized.

**Figure 2:**
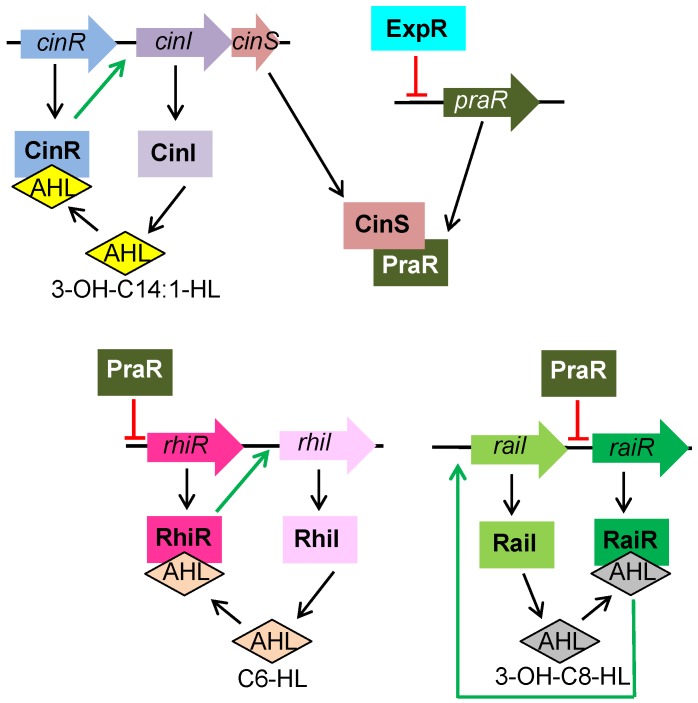
Quorum sensing regulatory circuit in *R. leguminosarum* bv. *viciae*. The Cin, Rhi and Rai systems are shown. The Cin system is at the top of the hierarchical regulatory cascade and is required for the activation of the Rhi and Rai systems through the action of the antirepressor CinS. The transcriptional repressor PraR is displaced by CinS at high population densities, allowing activation of the Rhi and Rai systems. ExpR also contributes to the activation of Rhi and Rai by repressing *praR* transcription. Genes are represented with wide arrows and proteins with rectangles. Green arrows represent activation; red flat-ended lines indicate repression. AHL: *N*-acyl homoserine lactone.

*R. leguminosarum* bv. *viciae* (Rlv), the symbiont of pea, vetch and lentil, possesses four different AHL-based QS systems with their LuxI-type AHL synthases (CinI, RhiI, RaiI, and TraI) and cognate LuxR-type regulators (CinR, RhiR, RaiR, and TraR) [17,80,81] (Table 1 and Figure 2). In addition, several orphan LuxR-type regulators have been identified such as BisR and ExpR. The Tra system and BisR regulate the transfer of the symbiotic plasmid in Rlv in response to CinI-made AHL from potential recipient cells. For details, readers are referred to [83]. CinI, CinR and ExpR are the orthologs of SinI, SinR and ExpR, respectively, in *S. meliloti*. In Rlv, the Cin system is at the top of a hierarchical regulatory cascade, controlling the expression of the other QS systems and acting as an overall switch potentially influencing many aspects of rhizobial physiology [79,82,83,84]. CinI produces 3-OH-C14:1-HL and CinR regulates the expression of *cinI* in response to the CinI-made AHL. RhiI produces C6-HL and RhiR strongly induces the expression of *rhiI* and the *rhiABC* operon in response to RhiI-made AHL. RaiI produces 3-OH-C8-HL and RaiR regulates the expression of *raiI* in response to the RaiI-made AHL [84]. Apart from regulating the production of 3-OH-C8-HL, no other phenotypes have been associated to the Rai system of Rlv. A distinguishing feature of QS regulation in Rlv is that gene regulation relies on the population density-dependent accumulation of an antirepressor: CinS [81]. CinS is a small protein encoded by a gene which is co-transcribed with *cinI*, and is required to activate the Rhi and Rai systems [80] (Figure 2). CinS has the ability to bind PraR, a transcriptional regulator that represses *rhiR* and *raiR* expression. The anti-repressor activity of CinS does not require AHL. Binding of CinS to PraR displaces the repressor from the *rhiR* and *raiR* promoters, thereby inducing their expression. Full activation of Rhi and Rai systems also requires ExpR, which represses transcription of the *praR* gene (Figure 2). In *S. meliloti* an ortholog of *praR* is present (named *phrR*), but not a *cinS* ortholog [81]. As in Rlv, ExpR represses the promoter of the *phrR* gene of *S. meliloti* upon binding of ExpR/AHL to this promoter [28].

In *R. etli* two different isolates have been investigated, which share orthologous genes with strains of *R. leguminosarum* (Table 1). *R. etli* strain CNPAF512 has two QS regulatory systems, CinIR and RaiIR, both of which control symbiosis [73,75]. In contrast, strain CFN42 has a complex but less well characterized QS system composed of three different LuxI-type AHL synthases (CinI, RaiI, and TraI) and four cognate LuxR-type regulators (CinR, RaiR, TraR1, and TraR2). In this strain, the structure of only one *N*-acyl-homoserine lactone synthesized by TraI, 3-oxo-C8-HL, has been solved, although AHLs produced by the other synthases have been detected and are under investigation. Recently, it has been found that CinR can activate *cinI* expression in the absence of its ligand, thereby demonstrating the complexity of the QS regulatory pathways in this bacterium [76].

## 5. Functions Regulated by Quorum Sensing in Rhizobia

Several studies have shown that QS regulation plays an important role regulating functions which are crucial for rhizobial survival as a free-living bacterium, as well as for different stages of the symbiotic interaction with the legume host. Processes such as growth, transfer of plasmids and symbiosis islands, regulation of EPS production, motility, biofilm formation, nodulation or nitrogen fixation can be influenced by QS with different effects depending on the rhizobial species (Table 1). The plethora of genes whose expression is modulated in an AHL-dependent manner in rhizobia has been unveiled in genome-wide transcriptomic analyses [29,30,88,90]. Here, we will focus on the role of QS on relevant traits such as EPS production and motility and we will discuss the relevance of this regulatory system in the establishment of symbiosis. For information about QS-controlled transfer of plasmids and symbiosis islands, readers are referred to more detailed studies [69,77,83,87,92,93,94].

### 5.1. Exopolysaccharide Production

In rhizobia such as Rlv and *S. meliloti*, QS regulation influences production and/or processing of different EPS. QS also controls expression of EPS-related genes in *S. fredii* NGR234 [88]. In Rlv *cinS* and *expR* positively regulate the expression of *plyB*, which encodes a glycanase that cleaves the acidic EPS. This EPS is required for infection of peas. However, mutations in *cinS* or *expR* which decrease or abolish expression of *plyB* result in an increase in biofilm formation but do not affect nodulation [80]. In *S. meliloti*, the synthesis of galactoglucan or EPS II, which involves participation of *exp/wge* gene-encoded proteins, is abolished in the absence of any of the genes of the ExpR/Sin system [36,37], leading to a characteristic dry colony phenotype as mentioned in the previous Section 2. Moreover, the ExpR/Sin system also controls the production of succinoglycan or EPS I, which requires proteins encoded by the *exo* genes [38]. Without a functional QS regulatory system, levels of the low molecular weight (LMW) form of EPS I, which is the essential fraction for nodule invasion, are severely affected. More recently, the ExpR/Sin regulatory system has been shown to control the synthesis of a novel mixed-linkage β-glucan (MLG) [91]. Expression of the *bgsBA* genes encoding the enzymes required for the synthesis of this polysaccharide is activated by an artificial increase in levels of the second messenger cdG, and is dependent upon the ExpR/SinI regulatory system. Bacterial invasion of developing nodules induced by *S. meliloti* requires the synthesis of the LMW form of at least one of the two symbiotically essential EPS, succinoglycan or EPS II. Moreover, the LMW fraction of EPS II is crucial for biofilm formation and contributes to alfalfa root colonization [95]. MLG is not required for nodulation or nitrogen fixation, although it might influence root colonization.

### 5.2. Bacterial Motility

Genome-wide transcriptomic analyses have shown that QS-deficient mutants exhibit increased expression of chemotaxis and motility genes [29,30,88]. This process has been well investigated in *S. meliloti*. In this bacterium, at high population densities, motility genes are repressed by binding of AHL-activated ExpR within the promoter region of the *visNR* operon, down-regulating its expression. Because VisNR are the master regulators of the flagellar regulon, this results in the decreased expression of this set of genes [43]. However, at low cell densities, the outcome of ExpR and AHL regulation is different, since ExpR activates the expression of genes involved in motility. It was found that high concentrations of AHLs were not sufficient to repress flagellar gene expression at low cell density, while they did when cells were at the late-log phase. These results indicate that, besides AHLs, additional phase-related factors are required to down-regulate motility genes [30].

By controlling the production of EPS, the QS system impacts the surface motility behavior of *S. meliloti* strains. Production of copious amounts of EPS II associated with cells harboring a functional ExpR/Sin system allows for a flagellum-independent surface spreading or sliding [96]. This type of motility is characterized by the passive movement of cells across the agar surface driven by the expansive forces of bacterial growth and facilitated by the production of EPS that reduces friction between cells and the surface. QS regulation of EPS II synthesis is also responsible for surfing, a type of bacterial translocation that requires flagella and which is mainly driven by physical or chemical effects created by the secreted EPS II [97,98]. Only the high molecular weight form of EPS II facilitates this surface spreading. Moreover, among the multiple AHLs produced by *S. meliloti*, only two AHLs species, C16:1- and 3-oxo-C16:1-HLs, affected this mode of surface translocation by up-regulating the expression of the positive regulator for EPS II synthesis WggR (ExpG) [97]. In contrast to EPS II-mediated sliding and surfing, ExpR is not required for swarming motility, a specialized mode of surface translocation, dependent on rotating flagella and characterized by the rapid and coordinated movement of multicellular groups of bacteria [99]. ExpR-deficient *S. meliloti* strains such as Rm1021 and GR4 can translocate over semisolid surfaces using swarming motility [96,100]. Although this finding demonstrates that a fully functional QS system is not necessary for swarming, it does not rule out a possible role for AHLs and/or population density in the regulation of swarming motility, as it has been shown for many bacteria. In fact, AHLs carrying a long-chain fatty acid moiety produced by the bean symbiont *R. etli* have a dual role in the swarming motility exhibited by this bacterium: as QS signals and as biosurfactants which promote surface translocation [74].

In different bacteria, type IV pili have been involved in twitching motility, a mode of surface translocation powered by the extension and retraction of pili. In *S. meliloti*, AHL-activated ExpR represses expression of *pilA1*, a gene belonging to a chromosomal cluster encoding proteins for type IVb pili of the Flp (fimbrial low-molecular-weight protein) subfamily. This cluster is responsible for the formation of unilateral and polar bundles in *S. meliloti* [35]. Twitching motility has not been reported in rhizobia. Therefore, a putative role of the ExpR/Sin system in controlling this behavior could not be established in *S. meliloti*. Nevertheless, the Flp-type pili were found to affect competitive ability for nodulation in alfalfa plants, perhaps by enhancing attachment to plant roots, before the onset of EPS production [35]. Interestingly, in *S. fredii* NGR234, chromosomal type IV genes were also down-regulated in a QS-dependent manner [88].

### 5.3. Role of Quorum Sensing in the Rhizobim-Legume Symbiosis

In general, the role of QS regulation in the *Rhizobium*-legume symbiosis remains puzzling. Although it is clear that population density-dependent control of gene expression affects important behaviors in rhizobia, AHL-mediated regulation is not always essential for symbiosis (Table 1). It is especially surprising that mutations in similar genes of different rhizobial species have different effects on the interaction with their legume hosts. The most severe effects in symbiosis have been described for QS mutants in *R. etli* and *Mesorhizobium tianshanense* (Table 1). In *R. etli* CNPAF512 mutations in *cinI* or *cinR* caused abnormal development of bacteroids in bean nodules, as well as decreased symbiotic nitrogen fixation [73]. Similarly, in *R. etli* CFN42 *cinR*, *raiR*, and *traI* mutants showed decreased nitrogen fixation activity [76]. Mutants of *M. tianshanense* in genes highly similar to *cinI* and *cinR* were defective in nodulation on *Glycyrrhiza uralensis,* showing decreased nodule formation efficiency or even a Nod^-^ phenotype, depending on the isolate [70,71]. In contrast, mutation in *cinI* or *cinR* in Rlv did not significantly affect symbiosis with pea [80]. Likewise, whereas *raiI* inactivation in *R. etli* led to increased nodulation on beans, no symbiotic defect was detected in a *raiI* mutant of Rlv [75,84]. Interestingly, in the *R. etli*–bean symbiosis the *cin* and *rai* systems are expressed *in planta*, whereas QS systems are repressed in Rlv or *S. meliloti* bacteroids [30,101], indicating that QS does not play a regulatory role in the latter bacteria once the *Rhizobium*-legume symbiosis is established. It was suggested that QS genes could be influenced by the host plant, playing a role in nodules of legumes in which terminal bacteroid differentiation does not occur [4]. Conversely, in symbiosis with the galegoid legumes (e.g. alfalfa and pea), where the bacteroids differentiate into a terminal form, QS regulation could not be relevant.

In *S. meliloti,* a functional QS system is required for efficient symbiosis with its host plant. Inoculation of *Medicago sativa* plants with a *sinI* mutant, unable to produce AHLs, led to a significant reduction in the total number of nodules per plant compared to that in the wild type, as well as a delay in the appearance of nitrogen-fixing nodules. In contrast, an *expR* deficient strain, which produces comparable levels of AHL, establishes symbiosis as efficiently as the wild type [24,40]. The reason for the symbiotic impairment exhibited by the *sinI* strain might be caused by the mutant’s inability to both repress the synthesis of flagella and produce AHLs [30]. Interestingly, a *sinI* mutant incapable of producing flagella regained the ability to establish efficient symbiosis. The presence of flagella might interfere with proper progression of infection threads or activate plant defenses that hamper the invasion process. 

By coordinating expression of important phenotypes such as EPS production and motility, QS regulation in rhizobia may facilitate the transition from free-living to symbiotic lifestyles. In the soil, when the number of cells is low, activation of functions such as motility might be advantageous in search for nutrients or the appropriate host. Likewise, expression of type IV pili when the cell density is low might help tight attachment to roots and colonization. It is known that QS influences biofilm formation in different rhizobia [66,86,91,95,102]. QS-mediated promotion of biofilm formation contributes to optimal root colonization which in turn could influence nodule formation efficiency and competitiveness [86,103]. As the population density increases at the expense of root exudates, QS regulation takes place and coordinated repression of flagellar and pili production and activation of EPS synthesis allows efficient plant invasion. At this stage, repression of flagella and pili might be important to avoid recognition and alerting plant defenses.

A recent observation represents a good example of the complexity of the role of AHL in the *Rhizobium*-legume symbiosis. In the broad host range strain *S. fredii* NGR234, the absence of AHLs triggers a mechanism that allows this bacterium to initiate the nodulation process regardless of the presence of *nod*-gene inducers [89]. This response is mediated by an increase in the copy number of the symbiotic plasmid, which leads to increased expression of all plasmid-borne symbiotic genes. This implies that single cells can initiate infection of root hairs even in the absence of host-specific flavonoids, which could be another key for broad host range. Finally, besides all the AHL-mediated responses in the bacterium, AHLs can also trigger plant responses that can impact the plant-microbe interaction, adding an additional level of complexity. This aspect is discussed in the next section.

## 6. Rhizobial AHL in Interkingdom Communication

Several studies indicate that AHLs not only mediate cell-to-cell communication between bacteria, but also can be recognized by eukaryotic hosts and induce diverse reactions in them (reviewed in [104]). The reaction of plants to AHLs is specific and depends on the type of AHL and on the plant species. In contrast to prokaryotic signal detection, the lactone ring does not need to be intact for plants to exhibit some or all of their responses to AHLs, a feature that could be exploited to distinguish between plant and bacterial responses to the signal molecules [105]. 

The first study analyzing the effects that AHLs cause in plants was based on a proteomic analysis performed on the model legume *Medicago truncatula* treated with nanomolar concentrations of 3-oxo-C12-HL produced by *Pseudomonas aeruginosa* or 3-oxo-C16:1HL produced by *S. meliloti* [106]. Accumulation of a large number of root proteins related to plant defense, stress responses, flavonoid synthesis, hormones, and regulatory functions was detected. Some specific proteins were differentially produced depending on AHL structure, indicating that the plant could distinguish between different AHLs.

Later studies performed using different plant species found that treatment with different pure AHLs could influence plant growth and/or plant-microbe interactions (reviewed in [104]). The concentration and length of the acyl chain are important attributes for the plant response. In general, AHLs with a short acyl chain promote plant growth, whereas long-chain AHLs (≥ C12) affect interaction of plants with microbes but not plant development [107]. 

*Arabidopsis thaliana* plants inoculated with *S. meliloti expR*^+^ showed increased resistance against *Pseudomonas syringae* pv. tomato (Pst), whereas reduced or no effect on resistance could be observed when plants were treated with ExpR-deficient or AttM lactonase-expressing strains, which show lower or lactone hydrolyzed signal production, respectively [108]. Likewise, inoculation with a *R. etli* strain, which produces oxo-C8-HSL, had no impact on plant resistance. *S. meliloti expR*^+^ also induced resistance in barley, wheat and tomato plants against several fungal pathogens [109]. The effects caused by *S. meliloti expR*^+^ on plant defense responses were associated with *N*-3-oxo-tetradecanoyl-homoserine lactone (3-oxo-C14-HL) since chemical application of this AHL increased plant resistance towards several pathogens [107,110]. In *Arabidopsis*, 3-oxo-C14-HL-induced resistance is caused by priming, a process in which plants are able to respond faster and/or more strongly to a pathogen challenge [110,111,112].

Interestingly, whereas inoculation of *Arabidopsis* plants with 3-oxo-C14-HL induces resistance, the same treatment in *M. truncatula* was found to increase total nodule number upon rhizobial inoculation [113]. The positive influence of 3-oxo-C14-HL on nodulation is host-specific since the effect was not observed in *M. sativa* (alfalfa) despite being nodulated by the same rhizobial species. The effect on nodulation, which was not associated with changes in flavonoid production, lateral root formation or root growth, relies on an ethylene-dependent, but autoregulation-independent mechanism since the effects were observed in the autoregulation mutant *sunn4* (super numeric nodules4) but not in the ethylene-insensitive *sickle* mutant [113]. A more recent study found that total nodule number of *M. truncatula* increases upon treatment with different long-chain AHLs (≥ 12 carbons). Most of these AHLs also increased the rate of nodulation. However, whereas the latter response required the integrity of the lactone ring, the effect of AHLs on total nodule number was also observed with the ring-opened AHL, indicating that this response is driven by plant responses to these signals rather than a QS-mediated effect by the bacteria [105]. The finding that a comparable increase in nodulation was achieved with L-homoserine, a product resulting from the cleavage of the amide bond present in AHLs, suggests that some responses to AHLs in *M. truncatula* probably require signal hydrolysis by plant acylases or related enzymes. AHL amidolysis by a plant-derived fatty acid amide hydrolase (FAAH) has been demonstrated to mediate growth regulatory effects of AHL on *A. thaliana* [114]. The fact that rhizobial AHLs can have an impact on non-legume plants should be taken into account when trying to understand how AHLs impact nodulation. Again, it raises the question of how AHLs impact symbiosis, i.e., through direct, specific pathways or/and through general improvements in plant health.

## 7. Role of the Legume Host in Quorum Sensing Regulation in Rhizobia

Since QS regulates bacterial behaviors which are crucial in the outcome of host-microbe interactions, it seems logical that eukaryotes have developed strategies to interfere with the cell-to-cell communication system. During this process, known as quorum quenching (QQ), the host interferes with the signaling pathway or manipulates AHLs.

Several studies have shown that root exudates of different plants contain low molecular weight compounds that affect QS regulation in bacteria. These molecules can act as agonists or antagonists of QS, enhancing or inhibiting, respectively, AHL-regulated phenotypes [115,116]. The production of these AHL-mimics probably modulates the function and composition of bacterial populations in the rhizosphere, shaping the plant rhizomicrobiome [117].

Gao et al. (2003) [115] found that the model legume *M. truncatula* can produce 15 to 20 separable compounds with the capacity to affect QS regulation based on AHL. The secretion of these signals changed with the plant age. Interestingly, significant changes in the amount and kinds of signal-mimic compounds were detected when *M. truncatula* seedlings were exposed to different AHLs, indicating that the plant has the potential to manipulate the behavior of the different bacteria it encounters actively and specifically [106].

The chemical identities of plant molecules that affect bacterial communication mediated by AHLs are mostly unknown. Although it seems logical that AHL-mimics bind to the autoinducer-binding domain of LuxR-type regulators and therefore they should be structurally close to AHLs, different studies indicate that these compounds can have a structure very different from that of AHL [118,119,120]. L-canavanine, an arginine analog, was found in alfalfa seed exudates as a compound that inhibits QS-regulation in *S. meliloti* [118]. Specifically, this compound inhibited expression of *exp* genes required for EPS II production. The observation that AHL-mimics produced by rice and bean plants can alter biofilm formation in plant-interacting bacteria, including rhizobia [119], suggests that these compounds could have a pivotal role during the first steps in the plant-bacterium interaction.

Recently, the production of QS-like molecules by peanut plants (*Arachis hypogaea*) has been examined [121]. Peanut is a legume crop nodulated by *Bradyrhizobium* spp. which synthesize long-chain AHL. Seed and root exudates of peanut plants contained QS-mimics that function similar to long-chain AHL and inhibitors of functions regulated by short-chain AHL. Although the nature of these signal molecules remains to be determined, these results could indicate that peanut plants select bacteria with QS regulation mediated by long-chain AHLs and disrupts the QS mechanisms of bacteria that communicate through short-chain AHLs.

Future research will determine the nature as well as the mechanism of action of QS-mimic compounds produced by legume hosts. This knowledge will help to elucidate if these plant signals serve to stimulate crucial events in the *Rhizobium*-legume symbiosis such as infection initiation and/or bacteroid formation and at the same time block undesired bacterial phenotypes that could harm plant health.

The plant host can also affect QS regulation by influencing the availability of AHLs in the rhizosphere. Zarkani et al. (2013) [108] found that when *S. meliloti* was co-cultivated with *Arabidopsis* plants, the amount of AHLs was reduced. In that study, the authors did not investigate if the negative effect exerted by the plant on AHL levels was caused by inhibition of AHL production, or by altering their stability or availability. To the best of our knowledge, enzymatic activities able to modify AHL signals have not yet been identified in legumes. However, the presence of FAAH in *Arabidopsis* plants [114], together with the finding that animals can produce lactonases and oxidoreductases that alter AHLs [122,123] makes it foreseeable that similar enzymes might also be present in legumes allowing them to manipulate the behavior of potential colonizers, including rhizobia.

## 8. Conclusions

AHLs serve as cell-to-cell signals both in the recognition of “self”, i.e., intraspecies signaling, and recognition of other species, interspecies signaling. This situation suggests that AHL-mediated signaling is involved in complex, dynamic signaling between multiple partners in its natural habitat—the rhizosphere. However, most of the research cited here has been limited to monocultures. Understanding AHL-signaling will require firstly a robust knowledge of the signaling mechanisms and secondly how these mechanisms perform in multi-species mixtures. Key to the perception and control of AHL signaling are the LuxR-like regulators, and this is apparent not only from studies on *S. meliloti*, but also the other rhizobia. Multiple studies have revealed that while several of the LuxR-like homologs play a pivotal role in the perception and production control of AHLs, others are regulated by AHLs but do not directly interact with AHLs as the activating ligand, and still others appear to have no relationship to AHLs. One possibility accounting for this situation is that some LuxR-like proteins perceive non-AHL signals from other species, e. g., plant hosts, and integrate these into the control of transcription. Thus, future research aimed at understanding signaling behavior in the rhizobia should include a focus on the LuxR-like proteins, their activating signals and their regulons. Also unknown is whether AHLs have an impact on survival independently of the LuxR-like proteins. One of the best known LuxR-like regulons is that of ExpR, which controls up to 9% of the transcriptome of *S. meliloti*. However, the vast majority of the regulated genes have no known function. So far, most of the research has been limited to EPS production and motility, which probably represents a small part of the ExpR regulon. Hence future research should also focus on the regulatory targets of LuxR-like proteins and their role during survival of free-living *S. meliloti* in the rhizosphere and during symbiosis with the plant hosts.

## Figures and Tables

**Table 1:** Quorum-sensing systems in symbiotic rhizobia.

Rhizobial Strain	QS Signal	Genes	Phenotypes Regulated	References
*Bradyrhizobium japonicum* *(B. diazoefficiens)*				
USDA110	Bradyoxetin	Unknown	*nod* gene regulation	[20,64]
	Isovaleryl-HL	*bjaI/bjaR_1_*	Unknown.	[21]
USDA290 and other strains	Non-characterized AHL	Unknown	Unknown	[65]
*Bradyrhizobium* spp.				
ORS278	Cinnamoyl-HL	*braI/braR*	Unknown	[22]
Peanut-nodulating strains	C6-HL, 3-oxo-C10-HL, 3-oxo-C12-HL, 3-oxo-C14-HL	Unknown	Motility, biofilm formation, cell aggregation ^a^	[66]
SR-6	C6-HL, 3-OH-C6-HL, C8-HL, C10-HL, 3-oxo-C10-HL, 3-oxo-C12-HL, 3-OH-C12-HL	Unknown	Nodulation ^b^	[67]
*Mesorhizobium loti*				
NZP2213	3-oxo-C6-HL, C8-HL, C10-HL	*mrlI2*	Unknown	[68]
	C12-HL	*mrlI1*	Nodulation	
R7A	3-oxo-C6-HL	*traI1/traR*	Symbiosis island transfer	[69]
	C4-HL, 3-oxo-C12-HL	Unknown	Unknown	
*Mesorhizobium tianshanense*				
CCBAU3306	3-oxo-C6-HL ^c^, 3-oxo-C8-HL ^c^	*mrtI/mrtR*	Nodulation (Nod^-^)	[70]
CCBAU060A	3-oxo-C6-HL ^c^, 3-oxo-C8-HL ^c^, 3-oxo-C12-HL ^c^	*mtqI/mtqR/* *mtqS*	Growth rate, nodulation	[71]
*Mesorhizobium huakuii*				
CCBAU21173	Peptide-related signal	Putative peptidase	Growth rate, biofilm formation, nodulation (Nod^-^)	[72]
*Rhizobium etli*				
CNPAF512	3-OH-(slc)-HL ^d^	*cinI/cinR*	Nitrogen fixation, symbiosome development, growth rate, swarming	[73,74]
	Short-chain AHLs ^c^	*raiI/raiR*	Nodulation	[75]
CFN42	C8-HL ^c^, 3-oxo-C6-HL ^c^, 3-oxo-C8-HL ^c^	*cinI/cinR* *raiI/raiR*	Nodulation, nitrogen fixation	[17,76]
	3-oxo-C8-HL, 3-OH-C8-HL ^c^	*traI/traR1/traR2*	Plasmid transfer, nitrogen fixation,	[17,76,77]
RT1	3-oxo-C8-HL, 3-OH-C14-HL	Unknown	Swarming, biofilm formation ^a^	[78]
*Rhizobium leguminosarum*				
bv. viciae	3-OH-C14:1-HL	*cinI/cinR/cinS*	Growth inhibition, regulation of EPS cleavage, biofilm formation	[79,80,81]
	C6-HL, C7-HL, C8-HL	*rhiI/rhiR*	Nodulation ^e^	[4,82]
	3-oxo-C8-HL, C8-HL	*trai/traR, bisR*	Plasmid transfer	[4,83]
	C6-HL, C7-HL, C8-HL, 3-OH-C8-HL	*raiI/raiR*	Unknown	[84]
	Unknown effector	*expR*	Regulation of EPS cleavage, biofilm formation	[80,81]
*Sinorhizobium fredii*				
SMH12	C8-HL, C14-HL 3-oxo-C8-HL	Unknown *traI/traR*	Biofilm formation Plasmid transfer	[85,86]
NGR234	3-oxo-C8-HL	*traI/traR*	Plasmid transfer, growth rate, sedimentation, motility, biofilm formation, regulation of EPS genes, regulation of the copy number of the symbiotic plasmid	[87,88,89]
	Non-characterized AHL ^c^	*ngrI/ngrR*		
*Sinorhizobium meliloti* *(Ensifer meliloti)*				
Rm1021	C12-HL, C14-HL, 3-oxo-C14-HL, C16-HL, 3-oxo-C16-HL, C16:1-HL, 3-oxo-C16:1-HL, C18-HL	*sinI/sinR, expR*	EPS production, surface translocation, regulation of motility genes, growth rate, nodulation	[16,24,25,26,28,34,36,37,38,39,40,43,90,91]
Rm41 ^f^	3-oxo-C8-HL	*traI/traR*	Plasmid transfer	[27]

^a^ Effects observed by adding synthetic AHLs. ^b^ Effects observed by adding purified extracts of AHLs. ^c^ Detected by using biosensors but not characterized by mass spectrometry analysis. ^d^ Saturated long chain 3-hydroxy-acyl-HL. ^e^ Only in a mutant strain already compromised for nodulation. ^f^ Rm41 also harbors the ExpR/Sin system of Rm1021
